# Diagnosis of coronary artery disease in patients with type 2 diabetes mellitus based on computed tomography and pericoronary adipose tissue radiomics: a retrospective cross-sectional study

**DOI:** 10.1186/s12933-023-01748-0

**Published:** 2023-01-23

**Authors:** Xiaolin Dong, Na Li, Chentao Zhu, Yujia Wang, Ke Shi, Hong Pan, Shuting Wang, Zhenzhou Shi, Yayuan Geng, Wei Wang, Tong Zhang

**Affiliations:** 1 Department of Radiology, Fourth Affiliated Hospital of Harbin Medical University, No. 37, YiYuan Street, NanGang District, Harbin, 150001 Heilongjiang China; 2 Department of Interventional and Vascular, Fourth Affiliated Hospital of Harbin Medical University, No. 37, YiYuan Street, NanGang District, Harbin, 150001 Heilongjiang China; 3 Shukun (Beijing) Network Technology Co., Ltd, Jinhui Building, Qiyang Road, Beijing, 100102 China; 4 The MRI Room, First Affiliated Hospital of Harbin Medical University, No. 23, YouZheng Street, NanGang District, Harbin, 150001 Heilongjiang China

**Keywords:** Type 2 diabetes mellitus, Coronary artery disease, Computed tomography

## Abstract

**Background:**

Patients with type 2 diabetes mellitus (T2DM) are highly susceptible to cardiovascular disease, and coronary artery disease (CAD) is their leading cause of death. We aimed to assess whether computed tomography (CT) based imaging parameters and radiomic features of pericoronary adipose tissue (PCAT) can improve the diagnostic efficacy of whether patients with T2DM have developed CAD.

**Methods:**

We retrospectively recruited 229 patients with T2DM but no CAD history (146 were diagnosed with CAD at this visit and 83 were not). We collected clinical information and extracted imaging manifestations from CT images and 93 radiomic features of PCAT from all patients. All patients were randomly divided into training and test groups at a ratio of 7:3. Four models were constructed, encapsulating clinical factors (Model 1), clinical factors and imaging indices (Model 2), clinical factors and Radscore (Model 3), and all together (Model 4), to identify patients with CAD. Receiver operating characteristic curves and decision curve analysis were plotted to evaluate the model performance and pairwise model comparisons were performed via the DeLong test to demonstrate the additive value of different factors.

**Results:**

In the test set, the areas under the curve (AUCs) of Model 2 and Model 4 were 0.930 and 0.929, respectively, with higher recognition effectiveness compared to the other two models (each p < 0.001). Of these models, Model 2 had higher diagnostic efficacy for CAD than Model 1 (p < 0.001, 95% CI [0.129–0.350]). However, Model 4 did not improve the effectiveness of the identification of CAD compared to Model 2 (p = 0.776); similarly, the AUC did not significantly differ between Model 3 (AUC = 0.693) and Model 1 (AUC = 0.691, p = 0.382). Overall, Model 2 was rated better for the diagnosis of CAD in patients with T2DM.

**Conclusions:**

A comprehensive diagnostic model combining patient clinical risk factors with CT-based imaging parameters has superior efficacy in diagnosing the occurrence of CAD in patients with T2DM.

**Supplementary Information:**

The online version contains supplementary material available at 10.1186/s12933-023-01748-0.

## Background

Diabetes is a metabolic disorder with a high prevalence worldwide [1], with type 2 diabetes mellitus (T2DM) being the most common and highly prevalent subtype [2, 3]. Despite advances in the diagnosis and treatment of T2DM, the risk of cardiovascular disease in patients with T2DM remains high [4, 5]. Furthermore, studies have shown that coronary artery disease (CAD) is the leading cause of death in patients with T2DM [6, 7], and that CAD progresses asymptomatically, only occasionally following a severe clinical course [8–10]. These findings suggest that the prevention of CAD in patients with T2DM is becoming an important public health concern. Therefore, we also need to develop new models to improve the early diagnosis of CAD in patients with T2DM to actively prevent the development and progression of CAD.

Coronary computed tomography angiography (CTA) is considered an important modality for the noninvasive diagnosis of CAD in patients with T2DM [11, 12]; therefore, some of the imaging parameters obtained by coronary CTA are increasingly becoming auxiliary indicators for the diagnosis and stratification of CAD. Computed tomography (CT)-based fractional flow reserve (CT-FFR), with invasive FFR as the gold standard, has been shown to be highly accurate in the diagnosis of CAD as an indicator of functional myocardial ischaemia [13–15]. Furthermore, coronary artery calcium is an important marker for identifying CAD [16], and a high coronary artery calcium score (CACS) has been found to be a predictor of CAD and is independently associated with the occurrence of cardiovascular events [17, 18]. Vascular inflammation is a major driver of atherosclerosis and plaque rupture [19], and pericoronary adipose tissue (PCAT) based on coronary CTA has been demonstrated in recent years as a new sensitive marker to indicate the degree of coronary inflammation, which can be used to identify and stratify CAD [20].The CRISP-CT study found that significantly higher PCAT attenuation (PCATA) around the left anterior descending artery (LAD) and right coronary artery (RCA) was an important risk factor for increased cardiac mortality [21]. PCATA has consequently been recognized as an important marker of increased cardiovascular risk. The onset of inflammation alters the composition of PCAT via various processes, such as fibrosis and microvascular remodelling [22, 23], and radiomics analysis captures these subtle structural remodelling processes to reflect permanent changes in adipose tissue [24]. Radiomics extracts multiple features embedded in medical images to establish machine learning models [24].

In recent years, an increasing number of studies have examined patients with T2DM, and a higher LAD-PCATA [25], plaque volume and mild calcification [26], and increased epicardial adipose tissue volume [27] have been found to improve the prediction of future cardiovascular events and cardiogenic death in patients with T2DM. A higher CACS has also been shown to be an independent predictor of CAD in patients with T2DM [28]. However, these studies have focused on future cardiac death and serious cardiovascular events, such as heart failure, and no studies have yet combined the data for the early identification of patients with T2DM and CAD. Therefore, we aimed to explore the risk factors associated with patients with T2DM and CAD and to identify patients with suspected CAD in T2DM by establishing a model combining clinical data, CT-based imaging parameters and PCAT-radiomics. The resultant data were used to establish an appropriate diagnostic model to monitor patients with T2DM, reduce the coronary burden and save their lives at an early stage.

## Methods

### Study population

**Fig. 1:**
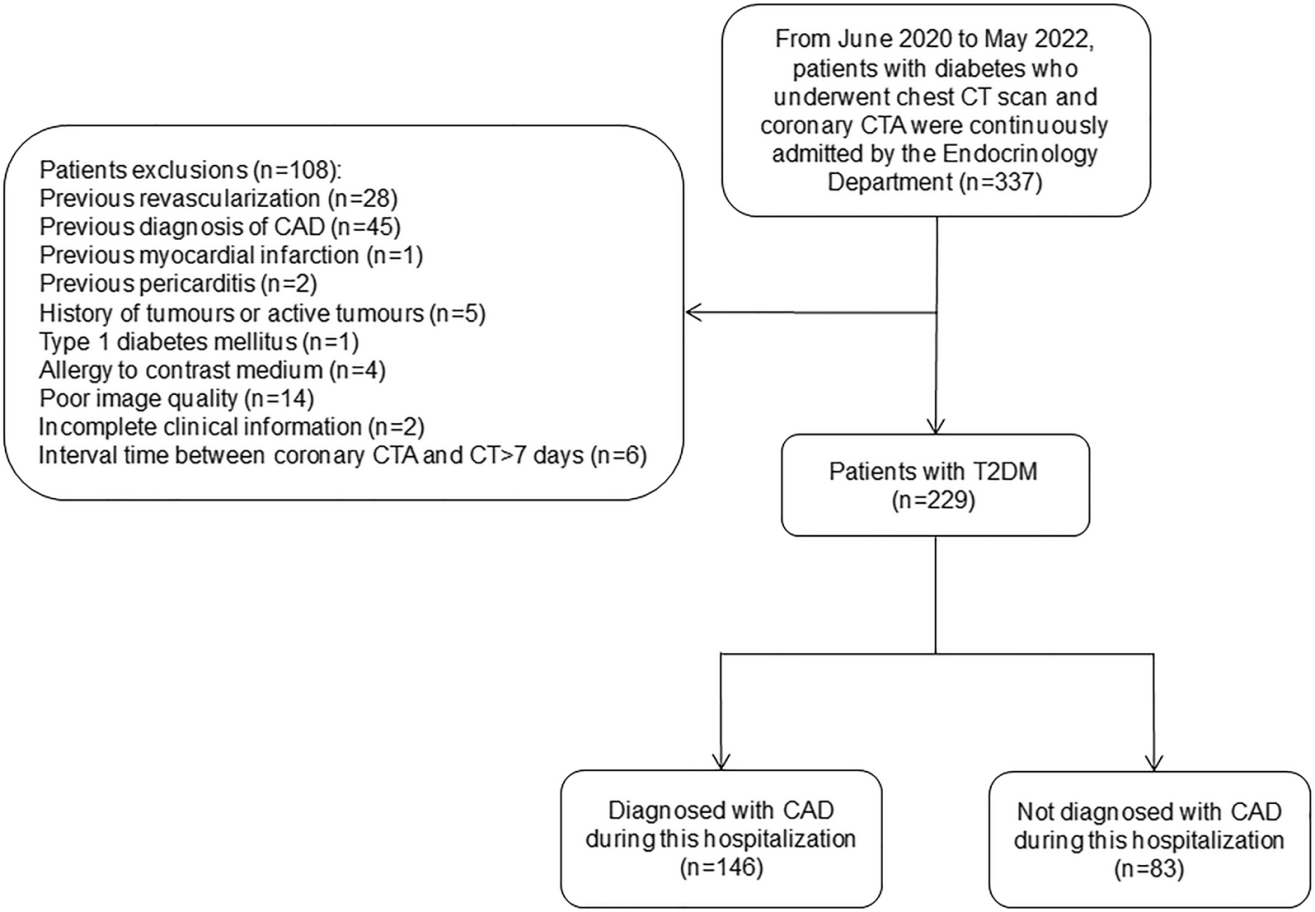
Flowchart showing inclusion and exclusion criteria for the study population. *CT* computed tomography, *CTA* computed tomography angiography, *CAD* coronary artery disease, *T2DM* type 2 diabetes mellitus

We retrospectively recruited patients with diabetes who were continuously admitted to the Department of Endocrinology at the Fourth Affiliated Hospital of Harbin Medical University from June 2020 to May 2022, including patients with previously diagnosed diabetes and those diagnosed during the current visit. A total of 337 patients underwent noncontrast chest CT and coronary CTA scans during this hospitalization for typical or atypical chest pain, and all had fasting blood samples taken for laboratory analysis within 24 h of admission. We excluded patients with previous coronary revascularisation (n = 28); previous cardiac diseases, including CAD (n = 45), myocardial infarction (n = 1) and pericarditis (n = 2); a history of tumours or active tumours (n = 5); type 1 diabetes mellitus (n = 1); contrast allergy (n = 4); poor image quality (n = 14); incomplete clinical information (n = 2); and coronary CTA with CT interval > 7 days (n = 6). Ultimately, 229 T2DM patients were included in this study, of whom 146 patients were diagnosed with CAD by invasive coronary angiography at this visit, including 109 patients with stable CAD and 37 patients with acute coronary syndrome; the remaining 83 patients did not develop CAD. Figure 1 illustrates the selection and exclusion of patients.

### Data collection

Two radiologists jointly collected the baseline features of the patients from clinical inpatient records, such as age, sex, body mass index (BMI), laboratory test data, previous medication use, CAD risk factors and diabetes-related complications. CAD, known as coronary atherosclerotic heart disease, is defined as heart disease caused by atherosclerosis of the coronary arteries that results in the narrowing or blockage of the lumen, leading to ischaemia, hypoxia or necrosis of the myocardium. CAD is divided into two main categories: stable CAD and acute coronary syndrome. Risk factors for CAD are classified according to the following criteria: (A) Hypertension: systolic blood pressure > 140 mmHg and/or diastolic blood pressure > 90 mmHg and/or use of antihypertensive medication according to the guidelines [29]; (B) Dyslipidaemia: dyslipidaemia defined as fasting total cholesterol > 6.2 mmol/L, low-density lipoprotein (LDL) cholesterol > 3.4 mmol/L, high-density lipoprotein (HDL) cholesterol < 1.0 mmol/L, serum triglycerides > 1.7 mmol/L (outpatient only), or diagnosis/treatment of dyslipidaemia [30]; (C) Diabetes mellitus: patients on oral hypoglycaemic agents or insulin or with fasting blood glucose ≥ 7.0 mmol/L are defined as T2DM [31]; (D) Family history of CAD: family history of ≥ 1 degree of consanguinity with CAD before age 60 [20]; (E) Obesity: BMI ≥ 28 kg/m^2^ is considered to be obese [32]. Diabetes-related complications include (A) retinopathy: any medical basis for retinopathy; (B) neuropathy: any sign or symptom of polyneuropathy; (C) peripheral arterial occlusive disease: 50% stenosis on ultrasonography; and (D) nephropathy: renal failure, i.e., glomerular filtration rate < 60 mL/min and/or micro or macro proteinuria [33, 34].

### CT image acquisition

All scans were performed on a 320-detector-row CT scanner (Aquilion ONE, Toshiba, Tokyo, Japan). The patients underwent a noncontrast chest CT on admission from the thoracic inlet to the abdomen. The patients also underwent coronary CTA scans during their hospital stay. All patients with a rapid heart rate at baseline were given an oral beta-blocker (25–75 mg) 1 h before the examination to reduce the heart rate to  ≤ 65 beats/min. A dual-channel high-pressure syringe was used to inject 60–80 mL of nonionic iodine contrast agent (iohexol injection containing 350 mg/L iodine, GE Healthcare, Beijing, China) at a flow rate of 4.5 mL/s, and coronary CTA image acquisition was performed using prospective ECG-triggered. The acquisition and reconstruction parameters in our study were as follows: 275 ms/time rack rotation speed, 120 kV tube potential, automatic adjustment of tube current according to patient BMI, 0.5 mm slice increments, images with 512 × 512 matrix and 0.25 mm or 0.5 mm increments. All scans were carried out during a single breath-hold. Two experienced cardiovascular imaging investigators interpreted the acquired imaging findings.

### Acquisition of image parameters and radiomic features

**Fig. 2:**
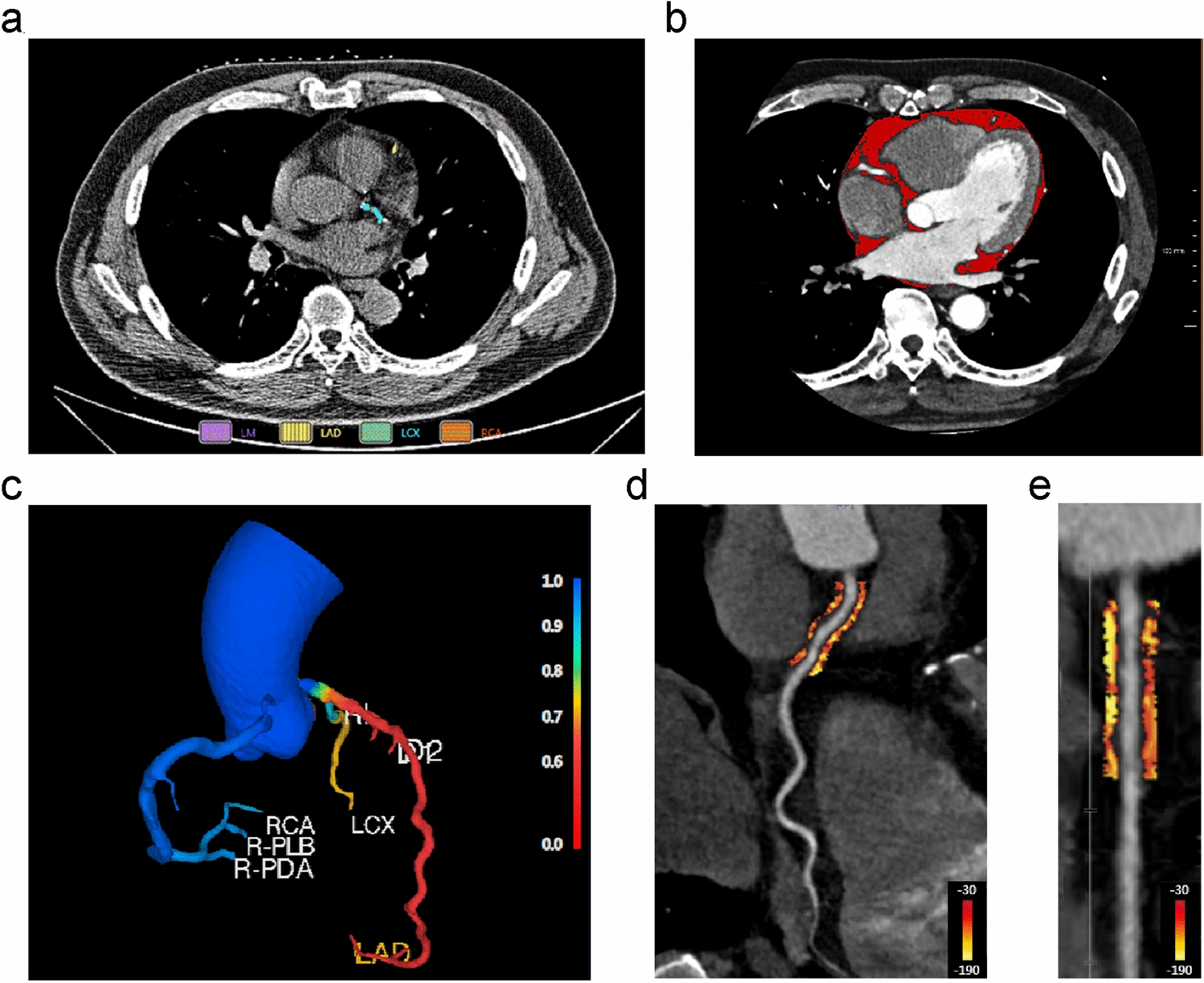
Representative case of imaging parameters measured by noncontrast chest CT and coronary CTA. **a** Example diagram of CACS measurements, yellow indicates calcification on LAD and green indicates calcification on LCX; **b** PAT volume measurement, red areas indicate pericardial adipose tissue; **c** CT-FFR model, CT-FFR values are marked in blue to red in the colour bar; **d** Curved multiplanar view showing PCAT quantification around the proximal coronary artery within reference diameter from the outer wall of the vessel, PCAT is visualized with adipose tissue HU colour table shown with colour bars; **e** shows the segment of the proximal coronary artery in a straightened view. *CT* computed tomography, *CTA* computed tomography angiography, *CACS* coronary artery calcium score, *PAT* pericardial adipose tissue, *CT-FFR* computed tomography-based fractional flow reserve, *PCAT* pericoronary adipose tissue

Based on noncontrast CT images [35], the CACS was calculated using the uAI Sphere (United Imaging Intelligence, v1.0.8214.7256) via the Agatston method [36], which is based on the principle of calcification density fractionation × calcification area (mm^2^). Plaques with CT values of 130–199, 200–299, 300–399 and ≥ 400 Hounsfield units (HU) were assigned a density score corresponding to 1, 2, 3 or 4, respectively. The scores for individual coronary arteries in all sections were summed to obtain the total coronary calcification score. The pericardial adipose tissue (PAT) volume was quantified using syngo.via (Siemens Healthcare GmbH, v06.07.0000.0000), which automatically calculates the total volume of tissue in the pericardial CT density range from − 190 HU to − 30 HU [37]. Using the semiautomatic quantification software CoronaryDoc^®^-FFR (Shukun (Beijing) Network Technology, v1.11.1), CT-FFR was calculated by combining the reduced-order method [38] and machine-learning modification [39]. Diameter stenosis (DS) was also quantified using the same software and was defined as (reference diameter−minimum lumen diameter)/reference diameter, patients with stenosis > 50% were considered to have significant stenosis [40]. PCAT was measured quantitatively using CoronaryDoc^®^. PCAT was defined as adipose tissue located within a radial distance from the outer vessel wall equal to the diameter of the coronary artery and with a CT attenuation range of − 190 HU to − 30 HU [21]. We tracked adipose tissue around three coronary trunks using an automated method, the 40 mm length range proximal to the LAD and left circumflex artery (LCX) and the 10 mm to 50 mm length segment proximal to the RCA. Figure 2 demonstrates an example of the abovementioned parameters. Moreover, we extracted 93 radiomic features from each PCAT (Additional file 1) from CoronaryDoc^®^. All imaging parameters were measured jointly by two radiologists, with a senior radiologist involved in case of disagreement.

### Model building and clinical application

**Fig. 3:**
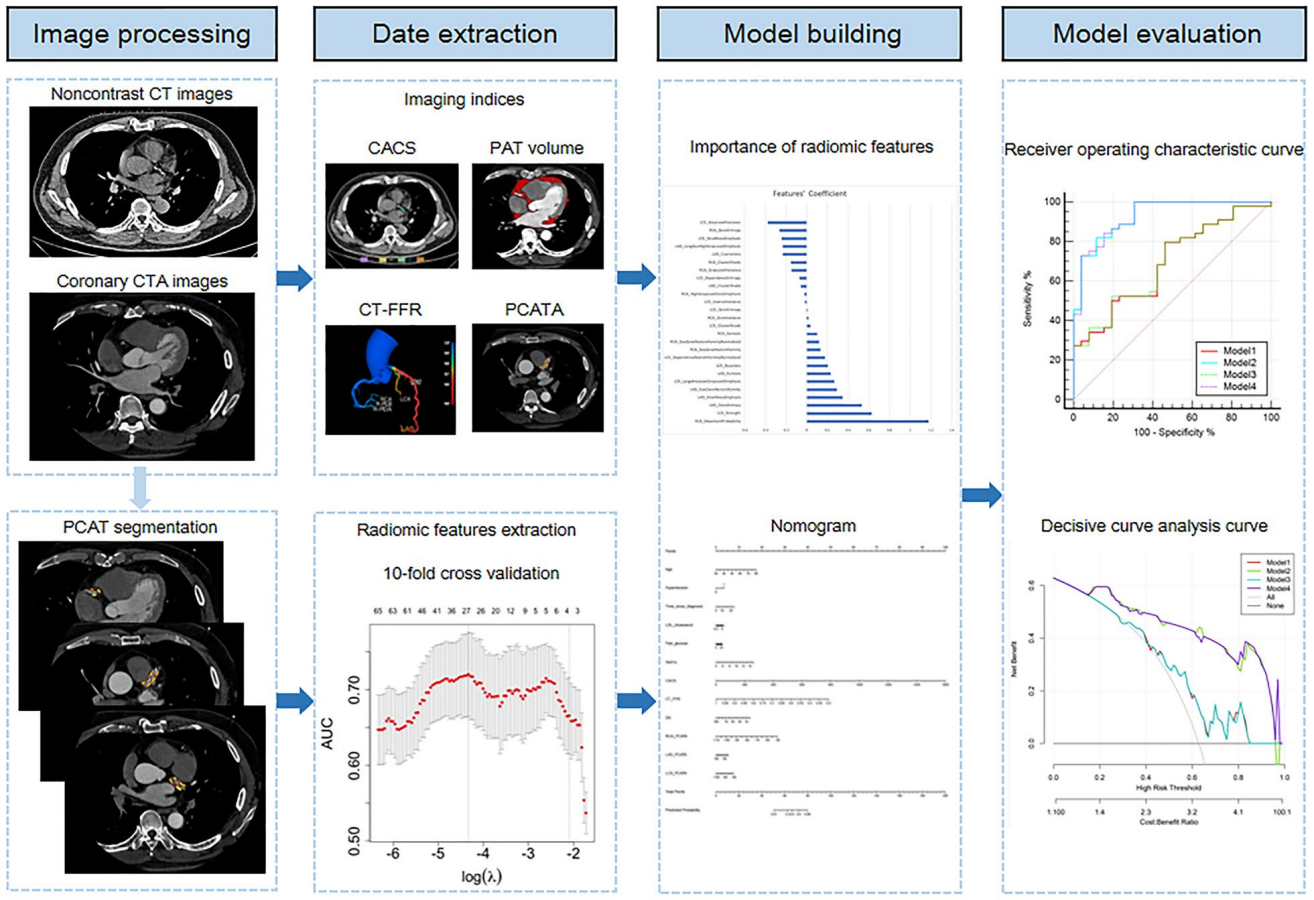
A flow chart of model development. *CTA* computed tomography angiography, *CT* computed tomography, *PCAT* pericoronary adipose tissue, *CACS* coronary artery calcium score, *PAT* pericardial adipose tissue, *CT-FFR* computed tomography-based fractional flow reserve, *PCATA* pericoronary adipose tissue attenuation

This study divided the dataset into training and test cohorts at a ratio of 7:3 at random. To select the most representative radiomic features, least absolute shrinkage and selection operator (LASSO) was applied, and the best lambda was determined with tenfold cross validation. Radscore [41] was added to other models to represent imaging features (detailed formula is listed in Additional file 1). Significant clinical factors and imaging indices were identified by univariate logistic regression with p < 0.05. Generalized logistic models were consecutively established with only clinical factors (Model 1), clinical factors and imaging indices (Model 2), clinical factors and Radscore (Model 3), and all together (Model 4) to examine their effect on discriminating patients with CAD from those without CAD. Sensitivity, specificity, accuracy, negative predictive value (NPV), positive predictive value (PPV) and area under the curve (AUC) were calculated to evaluate model performance. The DeLong test was performed to compare performance between models in a pairwise manner, and decisive curve analysis (DCA) was also plotted to assess clinical validity. The nomogram of the best model was illustrated according to the generalized logistic model. Figure 3 shows the workflow diagram for this study.

### Statistical analysis

The normality of continuous data was tested using the Shapiro‒Wilk test. Continuous variables are expressed as the mean ± standard deviation of normally distributed variables and median interquartile range of nonnormally distributed variables. Comparisons were made using Student’s t test or the Mann‒Whitney U test, as needed. Categorical variables are then expressed as absolute frequencies and percentages and were compared using the ϰ^2^ test or Fisher's exact test. Independent influences on patients with T2DM combined with CAD were identified using univariate and multivariate logistic regression analyses. A receiver operating characteristic (ROC) curve was used to evaluate the diagnostic efficacy of the four models for the diagnosis of CAD in patients with T2DM. A two-tailed p < 0.05 was considered statistically significant. R software (v4.1.0) and MedCalc (v20.115) were used for the statistical analysis and image plotting in this study.

## Results

### Patient characteristics

A total of 229 patients with T2DM were included in this study. The mean age of the patients was 58.79 ± 9.32 years, with 60.7% of patients being male. The clinical and imaging parameters of T2DM patients with and without CAD are shown in Table 1. Overall, 146 patients were diagnosed with CAD for the first time at this screening. Patients diagnosed with CAD were generally older (p < 0.001), more likely to have hypertension (p = 0.007) and dyslipidaemia (p = 0.008), and had higher levels of fasting glucose (p = 0.001) and glycated haemoglobin (HbA1c) (p < 0.001) than the remaining patients in the cohort. Remarkably, the time since T2DM diagnosis was longer in patients with concomitant CAD (p < 0.001). The CACS, CT-FFR, DS and PCATA significantly differed between patients with concomitant CAD and those without CAD (each p ≤ 0.001). The clinical and imaging characteristics of the patients in the training and test sets are shown in (Additional file 1: Table S1).

**Table 1:** Comparison of characteristics between T2DM patients with and without CAD

Characteristic	All	Patient with CAD	Patient without CAD	p
n	229	146	83	
Age (years)	58.79 ± 9.32	60.36 ± 9.01	56.02 ± 9.27	< 0.001
Male sex, n (%)	139 (60.7)	92 (63.0)	47 (56.6)	0.341
Body mass index (kg/m^2^)	26.00 [23.68; 27.70]	26.00 [23.60; 27.40]	25.70 [23.80; 28.10]	0.608
Obesity, n (%)	50 (21.8)	28 (19.2)	22 (26.5)	0.197
Smoking, n (%)	51 (22.3)	35 (24.0)	16 (19.3)	0.412
Drinking, n (%)	28 (12.2)	21 (14.4)	7 (8.4)	0.186
Hypertension, n (%)	142 (62.0)	100 (68.5)	42 (50.6)	0.007
Dyslipidaemia, n (%)	25 (10.9)	22 (15.1)	3 (3.6)	0.008
Family history, n (%)	20 (8.7)	15 (10.3)	5 (6.0)	0.274
Time since T2DM diagnosis (years)	7.00 [2.00; 12.25]	10.00 [4.00; 15.00]	3.00 [0.00; 8.00]	< 0.001
Aspirin, n (%)	12 (5.2)	9 (6.2)	3 (3.6)	0.405
Statin, n (%)	93 (40.6)	60 (41.1)	33 (39.8)	0.843
Metformin, n (%)	108 (47.2)	69 (47.3)	39 (47.0)	0.968
Insulin, n (%)	96 (41.9)	59 (40.4)	37 (44.6)	0.539
Oral antihyperglycemic drugs, n (%)	195 (85.2)	126 (86.3)	69 (83.1)	0.517
HDL-cholesterol (mg/dL)	0.99 [0.87; 1.12]	0.98 [0.86; 1.10]	1.02 [0.91; 1.17]	0.102
LDL-cholesterol (mg/dL)	3.43 [3.01; 3.79]	3.58 [3.09; 3.97]	3.28 [2.77; 3.53]	< 0.001
Total cholesterol (mg/dL)	5.28 [3.91; 6.00]	5.44 [3.99; 6.17]	4.66 [3.78; 5.78]	0.031
Triglyceride (mg/dL)	1.86 [0.65; 2.80]	0.83 [0.52; 2.72]	2.09 [0.93; 2.87]	0.003
Fast glucose (mmol/L)	9.60 [7.90; 12.21]	10.20 [8.30; 12.70]	8.80 [7.23; 11.28]	0.001
HbA1c (%)	7.70 [6.80; 9.10]	8.10 [7.40; 9.30]	7.00 [6.33; 8.43]	< 0.001
Retinopathy, n (%)	132 (57.6)	86 (58.9)	46 (55.4)	0.608
Neuropathy, n (%)	215 (93.9)	137 (93.8)	78 (94.0)	0.966
Peripheral arterial occlusive disease, n (%)	175 (76.4)	113 (77.4)	62 (74.7)	0.644
Nephropathy, n (%)	83 (36.2)	54 (37.0)	29 (34.9)	0.757
CACS	28.69 [4.15; 127.07]	70.68 [15.66; 224.41]	7.48 [1.61; 24.60]	< 0.001
PAT volume (mL)	178.44 [135.41; 230.29]	180.14 [138.64; 243.55]	173.55 [131.60; 212.33]	0.289
CT-FFR	0.83 [0.71; 0.90]	0.75 [0.64; 0.85]	0.90 [0.86; 0.94]	< 0.001
DS (%)	48.00 [22.00; 67.00]	57.50 [34.00; 72.00]	28.00 [0.00; 52.00]	< 0.001
RCA-PCATA (HU)	− 84.13 ± 9.64	− 80.94 ± 8.84	− 89.75 ± 8.38	< 0.001
LAD-PCATA (HU)	− 82.90 ± 8.59	− 80.50 [− 87.00; − 74.00]	− 86.00 [− 91.00; − 81.00]	< 0.001
LCX-PCATA (HU)	− 80.00 [− 87.00; − 74.00]	− 78.68 ± 8.06	− 84.00 ± 7.38	< 0.001

Values are mean ± standard deviation, median [25th and 75th percentile] or n (%). p values signify statistical significance and reflect the differences between patients with and without CAD. Obesity was defined as a body mass index ≥ 28 kg/m^2^

*T2DM* type 2 diabetes mellitus, *CAD* coronary artery disease, *HDL* high-density lipoprotein, *LDL* low-density lipoprotein, *HbA1c* glycated haemoglobin, *CACS* coronary artery calcium score, *PAT* pericardial adipose tissue, *CT-FFR* computed tomography-based fractional flow reserve, *DS* diameter stenosis, *PCATA* pericoronary adipose tissue attenuation

### Model establishment

Twenty-six radiomic features were selected as important features via LASSO and individual coefficients are plotted in (Additional file 1: Fig. S1). Six clinical factors and 6 imaging indicators were selected as important risk factors in univariate logistic regression (Table 2). Furthermore, multivariate logistic regression showed that age, HbA1c, CACS, CT-FFR, DS and RCA-PCATA were independent indicators in distinguishing CAD patients (each p < 0.05).

**Table 2:** Logistics regression analysis of factors associated with CAD

Characteristics	Univariable			Multivariable		
	OR	95%CI	p	OR	95%CI	p
Age	1.054	1.017–1.092	0.004	1.117	1.029–1.213	0.009
Gender	1.454	0.755–2.801	0.263			
BMI	0.990	0.904–1.084	0.832			
Obesity	0.664	0.313–1.408	0.286			
Smoking	1.350	0.623–2.927	0.447			
Drinking	1.935	0.669–5.594	0.223			
Hypertension	2.013	1.027–3.946	0.042	2.974	0.748–11.821	0.122
Dyslipidaemia	3.349	0.932–12.035	0.064			
Family history	3.667	0.791–17.003	0.097			
Time since T2DM diagnosis	1.145	1.076–1.219	< 0.001	1.086	0.973–1.212	0.138
Aspirin	1.719	0.335–8.81	0.516			
Statin	1.106	0.566–2.159	0.768			
Metformin	1.231	0.642–2.361	0.532			
Insulin	0.641	0.333–1.234	0.184			
Oral antihyperglycemic drugs	2.023	0.865–4.730	0.104			
HDL-cholesterol	0.327	0.083–1.290	0.110			
LDL-cholesterol	1.523	1.014–2.288	0.043	1.208	0.508–2.872	0.670
Total cholesterol	1.236	0.985–1.551	0.067			
Triglyceride	1.006	0.856–1.183	0.941			
Fast glucose	1.202	1.067–1.354	0.003	1.037	0.825–1.305	0.753
HbA1c	1.505	1.197–1.894	< 0.001	1.597	1.048–2.434	0.029
Retinopathy	1.407	0.734–2.697	0.303			
Neuropathy	1.208	0.326–4.470	0.778			
Peripheral arterial occlusive disease	1.821	0.843–3.933	0.127			
Nephropathy	0.976	0.498–1.912	0.943			
CACS	1.021	1.010–1.031	< 0.001	1.020	1.006–1.034	0.003
PAT volume	1.004	1.000–1.008	0.055			
CT-FFR	0.000	0.000–0.000	< 0.001	0.000	0.000–0.000	< 0.001
DS	1.038	1.024–1.052	< 0.001	0.954	0.918–0.993	0.018
RCA-PCATA	1.100	1.052–1.149	< 0.001	1.151	1.052–1.260	0.002
LAD-PCATA	1.042	1.003–1.083	0.036	0.964	0.88–1.054	0.424
LCX-PCATA	1.078	1.031–1.127	0.001	1.063	0.956–1.181	0.260

*CAD* coronary artery disease, *OR* odds ratio, *95%CI* 95% confidence interval, *T2DM* type 2 diabetes mellitus, *HDL* high-density lipoprotein, *LDL* low-density lipoprotein, *HbA1c* glycated haemoglobin, *CACS* coronary artery calcium score, *PAT* pericardial adipose tissue, *CT-FFR* computed tomography-based fractional flow reserve, *DS* diameter stenosis, *PCATA* pericoronary adipose tissue attenuation

### Model performance and pairwise comparison

**Fig. 4:**
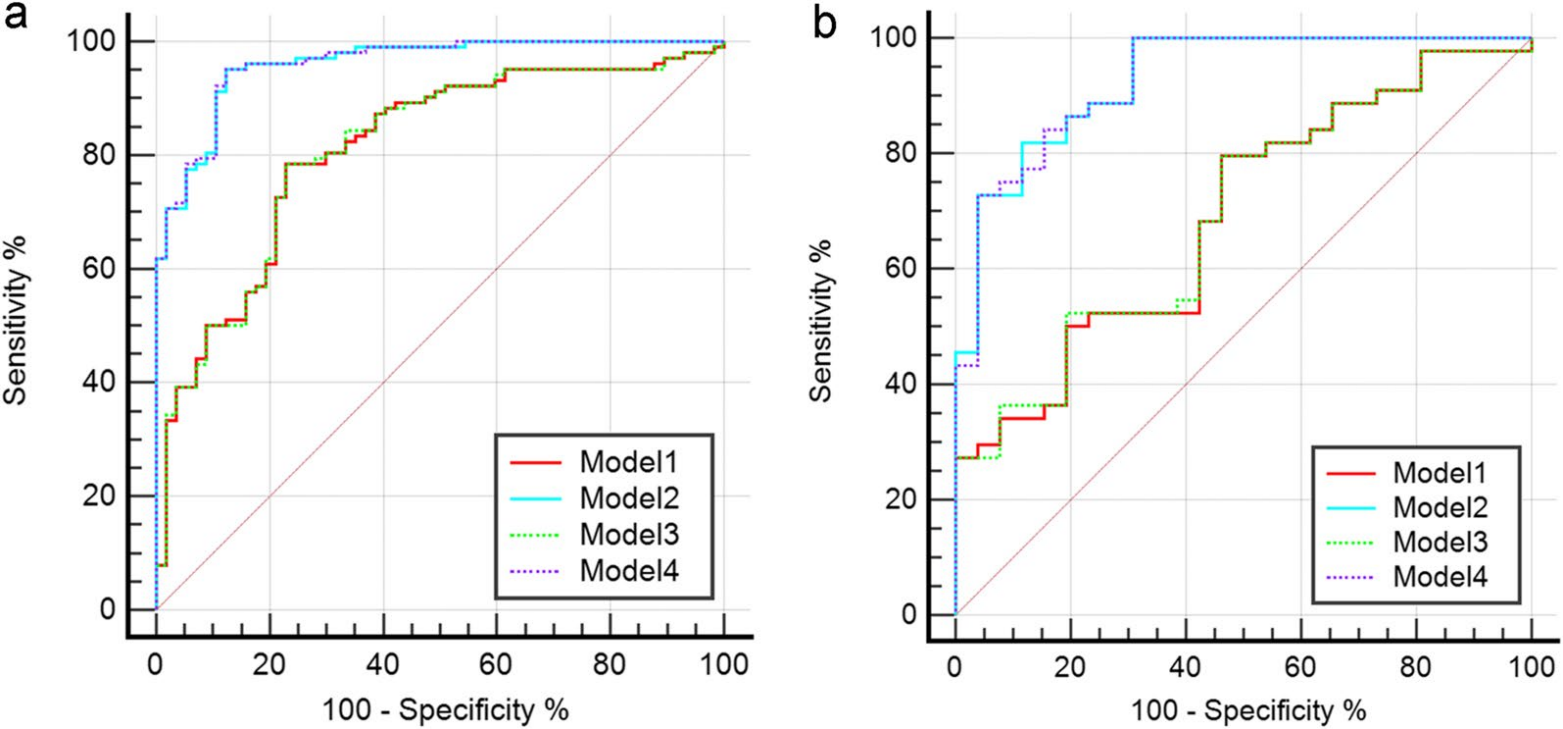
Receiver operating characteristic curves of all models in the training **a** and test **b** sets. *Model 1* clinical factors model, *Model 2* clinical factors and imaging indexes model, *Model 3* clinical factors and Radscore model, *Model 4* combined model

**Fig. 5:**
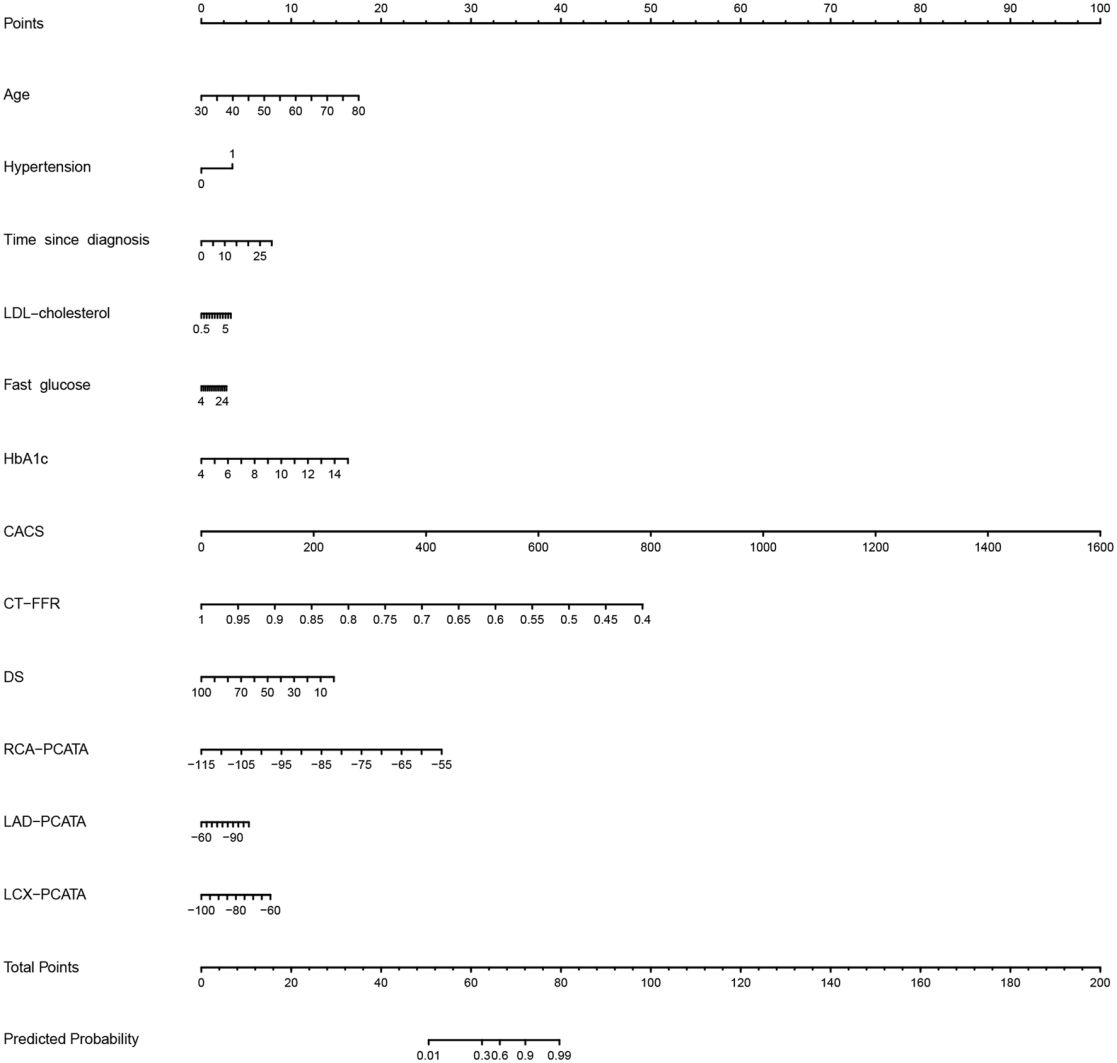
The nomogram of Model 2. The sum of all factors equals the total points. *Model 2* clinical factors and imaging indexes model, *LDL* low-density lipoprotein, *HbA1c* glycated haemoglobin, *CACS* coronary artery calcium score, *CT-FFR* computed tomography-based fractional flow reserve, *DS* diameter stenosis, *PCATA* pericoronary adipose tissue attenuation

**Fig. 6:**
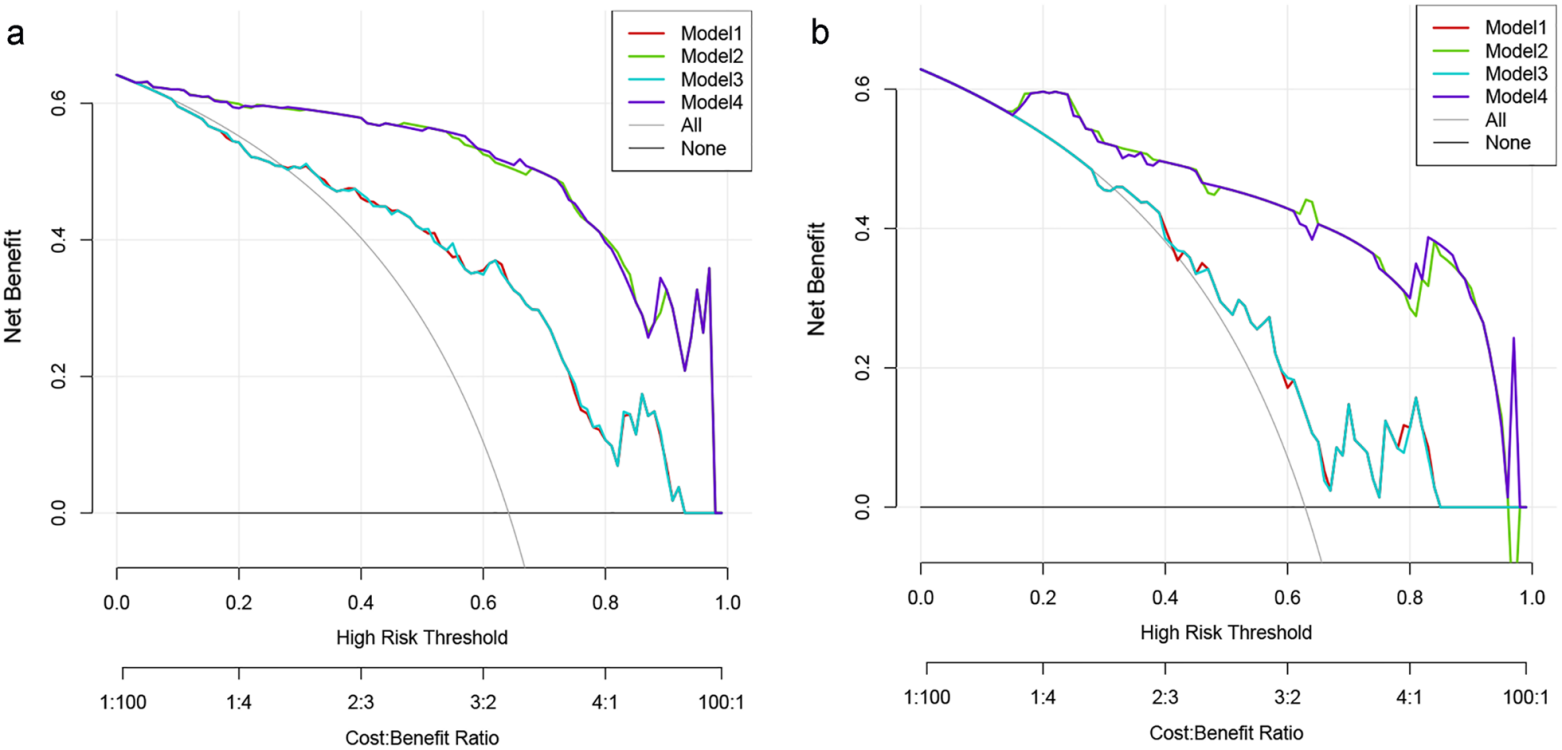
Decision curve analysis for the four models in the training **a** and test **b** sets. *Model 1* clinical factors model, *Model 2* clinical factors and imaging indexes model, *Model 3* clinical factors and Radscore model, *Model 4* combined model

The ROC curves of the four models in the training and test sets are plotted in Fig. 4. Models 1 and 3 were quite similar in performance, and the same trend appeared for Models 2 and 4*.* Table 3 shows the detailed model performance parameters, such as AUC, sensitivity, specificity, accuracy, PPV and NPV, in both datasets. Models 2 and 4 achieved the most encouraging results, with AUCs of 0.960 [0.934–0.987] and 0.930 [0.871–0.989] for Model 2 and 0.961 [0.934–0.988] and 0.929 [0.869–0.989] for Model 4 in the training and test datasets, respectively; both models produced significantly different results than Models 1 and 3 (each p < 0.001, DeLong test, detailed results in Additional file 1: Table S2). The diagnostic value of Model 2 for CAD was markedly higher than that of Model 1, with a significant difference between the AUCs of the two models (p < 0.001, 95% CI [0.129–0.350]). Notable, adding the Radscore to Models 1 and 2 did not significantly improve the recognition performance of the models (each p > 0.05). Model 2 can be further developed as a quantitative tool for the subsequent identification of the probability of CAD occurrence in patients with T2DM, which we present in the form of a nomogram (Fig. 5). The DCA curve indicated that Models 2 and 4 could add more benefit than Models 1 and 3 (Fig. 6).

**Table 3:** Recognition ability of all models for patients with CAD

Model	Training set						Test set					
	AUC (95%CI)	SEN	SPE	ACC	PPV	NPV	AUC (95%CI)	SEN	SPE	ACC	PPV	NPV
Model 1	0.811(0.741–0.881)	0.784	0.772	0.780	0.667	0.860	0.691(0.564–0.817)	0.577	0.659	0.629	0.500	0.725
Model 2	0.960(0.934–0.987)	0.951	0.877	0.925	0.909	0.933	0.930(0.871–0.989)	0.885	0.773	0.814	0.697	0.919
Model 3	0.812(0.742–0.882)	0.784	0.772	0.780	0.667	0.860	0.693(0.567–0.819)	0.577	0.659	0.629	0.500	0.725
Model 4	0.961(0.934–0.988)	0.951	0.877	0.925	0.909	0.933	0.929(0.869–0.989)	0.885	0.773	0.814	0.697	0.919

*AUC* area under curve, *95%CI* 95% confidence interval, *SEN* sensitivity, *SPE* specificity, *ACC* accuracy, *PPV* positive predictive value, *NPV* negative predictive value

*Model 1* clinical factors model

*Model 2* clinical factors and imaging indexes model

*Model 3* clinical factors and Radscore model

*Model 4* combined model

### Effect of the degree of stenosis

We grouped all patients with T2DM according to whether significant stenosis occurred in the coronary arteries. Patients with T2DM with significant stenosis had higher CACS (p < 0.001), lower CT-FFR (p < 0.001) and higher PCATA around the three coronary arteries (p < 0.05), but the PAT volume did not significantly differ between the two groups (p = 0.325) (Additional file 1: Table S3). The same results were obtained for patients with T2DM without significant stenosis (Additional file 1: Table S4). However, when patients with T2DM with CAD were divided into two groups based on the presence or absence of significant stenosis, the RCA-PCATA significantly differed between groups (p = 0.020), whereas the LAD-PCATA and LCX-PCATA did not significantly differ between groups (each p > 0.05) (Additional file 1: Table S5). Compared with patients without CAD, patients with T2DM with CAD demonstrated more serious disease progression, as evidenced by the CACS, CT-FFR and RCA-PCATA, irrespective of the presence of significant stenosis, but patients with significant stenosis had a heavier coronary burden than those without significant stenosis.

## Discussion

In this study, four models were developed to diagnose CAD in patients with T2DM, and their performance was validated in all aspects. Both the model combining clinical risk characteristics and CT imaging parameters as well as the combined model were found to be superior in identifying the occurrence of CAD, but Model 4, which added the Radscore to Model 2, did not improve the diagnostic performance compared to Model 2. We also developed a nomogram to diagnose patients with T2DM with CAD for future clinical applications. Additionally, patients with T2DM with CAD have a higher coronary burden, regardless of whether significant coronary stenosis has occurred.

Patients with T2DM have higher levels of chronic inflammation in the coronary vasculature because the high glycaemic state of the blood promotes the infiltration of macrophages and T cells [40, 42], causing aggregation of foam cells [43], while vascular inflammation leads to increased concentrations of circulating cytokines thus leading to atherosclerosis [44]. Therefore, T2DM is considered an independent risk factor for atherosclerosis that increases the risk of cardiovascular disease [26]. The T2DM population is considered to be a heterogeneous group of patients with and without CAD [28], and CAD is gaining attention as a major cause of death in T2DM. Therefore, we plan to develop a model to identify patients with T2DM and concomitant CAD. Previous studies have shown that the CACS can be used as a proxy for coronary atherosclerotic plaque burden to predict cardiovascular risk [45], and the CACS increases with cardiovascular risk in the T2DM patient population [46] to serve as a composite measure of atherosclerotic disease [16]. CT-FFR, as an indicator of coronary vascular haemodynamic stenosis, provides both physiological and corresponding anatomical information about the coronary vessels [47]; DS directly reflects the anatomical stenosis of the coronary vessels. Whereas PAT is located near the myocardium, and its excessive activity accelerates coronary atherosclerosis and, thus, cardiovascular disease [48]. However, logistic regression analysis revealed that PAT volume was not an influential factor in the development of CAD in patients with T2DM, and we speculate that this lack of influence may be because PAT is not in direct contact with the myocardium; therefore, PAT is not directly influenced by paracrine signals from the myocardium [23], which is similar to the findings of a previous study [27]. As adipose tissue is closer to the coronary arteries, there is a closer bidirectional influence between PCAT and the coronary vessel wall [49]; inflammatory changes in the coronary arteries affect alterations in the water–lipid balance in PCAT [49], causing dynamic changes in PCATA and similarly in high-dimensional features, and the discovery of such alterations in fine structure relies on the application of radiomics [24, 50]. In particular, age and HbA1c levels were independently associated with the development of CAD among the patients' clinical risk factors by multivariate logistic regression analysis. We assume that this result may be because older patients have more severe cardiovascular ageing and consequently have a more severe cardiovascular risk [51]. Moreover, HbA1c indicates the patient’s glycaemic control, and poor glycaemic control (HbA1c ≥ 7.0%) can lead to endothelial dysfunction, which negatively impacts the coronary arteries [52].

Our study used logistic regression to screen for clinical factors and CT imaging indices associated with the development of CAD and additionally combined the radiomic parameters of PCAT to develop a model to identify patients with T2DM and concomitant CAD. This study is the first to combine multiple factors to diagnose the occurrence of CAD in patients with T2DM. While previous studies have focused on the future occurrence of late cardiovascular events in patients with T2DM [25–27, 45], we have focused on CAD with the aim of detecting the onset of CAD before an irreversible endpoint event occurs in patients with T2DM. We have developed an optimal model nomogram to help patients proactively monitor the progression of their disease and the probability of CAD based on their condition and examination findings to proactively prevent and treat CAD in a timely manner. Alternatively, previous studies have reported that anti-inflammatory interventions and statin therapy result in a significant reduction in PCATA [53, 54], which may affect the subsequent clinical performance of patients with T2DM. Thus, we also collected patients' medication history, including not only aspirin and statins but also various types of hypoglycaemic drugs. Regrettably, these drugs were not found to be associated with the development of CAD when the initial parametric screening was performed.

Our study combined a variety of CT imaging indices based on clinical factors. First, the Agatston-based CACS was included in the model, and previous studies have also identified the CACS as a risk factor for the development of CAD in patients with T2DM [28], mainly because the CACS, which is based on direct measurements of the Agatston score, represents the extent and severity of atherosclerosis [55], which provides a direct response to coronary artery calcification. Whereas CT-FFR and DS reflect functional stenosis and anatomical stenosis of the coronary arteries, respectively, CT-FFR has also been shown in the past to provide greater accuracy in predicting coronary haemodynamic lesions [39]. In contrast, PCATA has been more extensively studied, and the value of a higher PCATA in predicting future cardiovascular events in patients with T2DM has been demonstrated [25]. Conversely, we found that RCA-PCATA was more heavily represented than LAD and LCX in the model for identifying CAD and that it was an independent influence on CAD occurrence, possibly because compared to the other two coronary trunks, there is less interference near the RCA, the measurements are more accurate, and the correlation with coronary inflammation has been validated at the molecular biological level [49]. Yu et al. also suggest that RCA-PCATA can be used to capture early inflammation around the coronary arteries [40]. Unexpectedly, however, the addition of radiomics to either Model 1 or 2 did not improve the model's identification efficacy. We suspected that the reason for this lack of improvement is that in patients with T2DM, the coronary arteries have already undergone a certain degree of inflammatory changes, which affect the spatial distribution of voxel intensity of PCAT in CTA images [56]. Furthermore, the effect on the spatial position of voxels in the images may be weakened when CAD occurs on this basis. Second, CT parameters that reflect functional and anatomical changes in the coronary arteries may be more valuable for the diagnosis of CAD in patients with T2DM than radiomics. Furthermore, we found that more than half of the patients were diagnosed with CAD, which we believe may be because diabetes is an independent risk factor for cardiovascular disease, and indeed, patients with T2DM have a very high probability of cardiovascular events; moreover, coronary CTA is not a routine test, and some of the patients with T2DM who participated in this test had symptoms or signs related to CAD detected during hospitalization, either clinically or by electrocardiogram, and thus using coronary CTA to supplement diagnosis.

We have developed a more comprehensive model for diagnosing CAD in patients with T2DM that incorporates the patient's clinical risk factors and CT imaging parameters. The nomogram created by this model captures the probability of CAD risk in patients with T2DM and allows for a noninvasive method of discerning the existence of CAD in patients. Thus, this model allows for a more proactive approach to prevent development and progression of CAD. As a noninvasive rapid diagnostic aid, the model can help clinicians and patients with T2DM to monitor and identify CAD early and in real time during the subclinical period before it progresses to have clinical manifestations. Clinicians can then aggressively deploy treatment to reduce the cardiovascular burden and risk of death for patients due to CAD. Treatment may even be implemented before the development of CAD, promoting the evolution of precision medicine. The measurements of the imaging parameters in this study were fully automated, which greatly improves the reproducibility of the study. Our patient cohort was smaller than those in previous studies [57, 58], but we collected more comprehensive clinical information than previous studies, measured a more comprehensive range of CT imaging parameters, and combined these data with radiomic parameters that have emerged in recent years to obtain a more efficient model.

This study was subject to several limitations that need to be addressed. First, this work was a single-centre retrospective study, and all patients used the same CT scan protocol with some selection bias. Therefore, more external validation is needed to improve the generalizability of the model. Second, our study demonstrates that the occurrence of T2DM with CAD is independently correlated with some clinical and CT imaging parameters but does not suggest a direct causal relationship between these parameters. Third, our study is a cross-sectional observational study, and we currently cannot describe the changes in the PCATA, CT-FFR and CACS over the course of the patients' disease. The next step is to collect relevant data for follow-up observation. Fourth, we can only know the type of medication used by the patients in the past but not the duration of medication use. Finally, some patients were restricted from receiving coronary CTA due to severe renal insufficiency, and this group of patients is consequently not represented in our study. In the future, we will consider conducting relevant studies mainly in patients with renal insufficiency.

## Conclusions

Our study shows that a model based on clinical features and CT imaging indices has the highest efficacy in diagnosing the occurrence of CAD in patients with T2DM. Our findings may help clinicians and patients with T2DM monitor and rapidly identify CAD in real time, provide timely diagnosis and treatment, and reduce future deaths due to cardiovascular disease.

## Supplementary Information

**Additional file 1. Table S1.** Baseline characteristics of the training set and test set. **Table S2.** Pairwise comparison of the AUCs of the test set by the DeLong test. **Table S3.** Comparison of CT parameters in patients with T2DM with significant coronary stenosis. **Table S4.** Comparison of CT parameters in patients with T2DM without significant coronary stenosis. **Table S5.** Comparison of CT parameters in patients with CAD with and without significant stenosis. **Figure S1.** The relative importance of radiomics features.

## Data Availability

The datasets used and/or analysed during the current study are available from the corresponding author on reasonable request.

## Abbreviations

T2DM: Type 2 diabetes mellitus
CAD: Coronary artery disease
CTA: Computed tomography angiography
CT: Computed tomography
CT-FFR: Computed tomography-based fractional flow reserve
CACS: Coronary artery calcium score
PCAT: Pericoronary adipose tissue
PCATA: Pericoronary adipose tissue attenuation
LAD: Left anterior descending artery
RCA: Right coronary artery
BMI: Body mass index
LDL: Low-density lipoprotein
HDL: High-density lipoprotein
HU: Hounsfield units
PAT: Pericardial adipose tissue
DS: Diameter stenosis
LCX: Left circumflex artery
LASSO: Least absolute shrinkage and selection operator
AUC: Area under the curve
NPV: Negative predictive value
PPV: Positive predictive value
DCA: Decisive curve analysis
ROC: Receiver operating characteristic
HbA1c: Glycated haemoglobin
