# Supplementary material for: Diagnosis of coronary artery disease in patients with type 2 diabetes mellitus based on computed tomography and pericoronary adipose tissue radiomics: a retrospective cross-sectional study
Source: Cardiovasc Diabetol. 2023 Jan 23;22:14. doi: 10.1186/s12933-023-01748-0 (PMC9869509; doi:10.1186/s12933-023-01748-0)
Supplement: Supplementary file 1 — Additional file 1. Table S1. Baseline characteristics of the training set and test set. Table S2. Pairwise comparison of the AUCs of the test set by the DeLong test. Table S3. Comparison of CT parameters in patients with T2DM with significant coronary stenosis. Table S4. Comparison of CT parameters in patients with T2DM without significant coronary stenosis. Table S5. Comparison of CT parameters in patients with CAD with and without significant stenosis. Figure S1. The relative importance of radiomics features. [file 12933_2023_1748_MOESM1_ESM.docx]

**Additional file 1**

**Methods**

Acquisition of image parameters and radiomic features

Radiomic features extracted from each PCAT

RadiomicsFirstOrder: 10Percentile, 90Percentile, Energy, Entropy, InterquartileRange, Kurtosis, Maximum, MeanAbsoluteDeviation, Mean, Median, Minimum, Range, RobustMeanAbsoluteDeviation, RootMeanSquared, Skewness, TotalEnergy, Uniformity, Variance;

RadiomicsGLCM: Autocorrelation, ClusterProminence, ClusterShade, ClusterTendency, Contrast, Correlation, DifferenceAverage, DifferenceEntropy, DifferenceVariance, Id, Idm, Idmn, Idn, Imc1, Imc2, InverseVariance, JointAverage, JointEnergy, JointEntropy, MCC, MaximumProbability, SumAverage, SumEntropy, SumSquares;

RadiomicsGLSZM: GrayLevelNonUniformity, GrayLevelNonUniformityNormalized, GrayLevelVariance, HighGrayLevelZoneEmphasis, LargeAreaEmphasis, LargeAreaHighGrayLevelEmphasis, LargeAreaLowGrayLevelEmphasis, LowGrayLevelZoneEmphasis, SizeZoneNonUniformity, SizeZoneNonUniformityNormalized, SmallAreaEmphasis, SmallAreaHighGrayLevelEmphasis, SmallAreaLowGrayLevelEmphasis, ZoneEntropy, ZonePercentage, ZoneVariance;

RadiomicsGLRLM: GrayLevelNonUniformity, GrayLevelNonUniformityNormalized, GrayLevelVariance, HighGrayLevelRunEmphasis, LongRunEmphasis, LongRunHighGrayLevelEmphasis, LongRunLowGrayLevelEmphasis, LowGrayLevelRunEmphasis, RunEntropy, RunLengthNonUniformity, RunLengthNonUniformityNormalized, RunPercentage, RunVariance, ShortRunEmphasis, ShortRunHighGrayLevelEmphasis, ShortRunLowGrayLevelEmphasis;

RadiomicsNGTDM: Busyness, Coarseness, Complexity, Contrast, Strength;

RadiomicsGLDM: DependenceEntropy, DependenceNonUniformity, DependenceNonUniformityNormalized, DependenceVariance, GrayLevelNonUniformity, GrayLevelVariance, HighGrayLevelEmphasis, LargeDependenceEmphasis, LargeDependenceHighGrayLevelEmphasis, LargeDependenceLowGrayLevelEmphasis, LowGrayLevelEmphasis, SmallDependenceEmphasis, SmallDependenceHighGrayLevelEmphasis, SmallDependenceLowGrayLevelEmphasis.

Model building and clinical application

Radscore detailed formula

Radscore = RCA_MaximumProbability×1.178+LCX_Strength×0.628+LAD_ZoneEntropy×0.531+LAD_SmallAreaEmphasis×0.345+LAD_SizeZoneNonUniformity×0.290+LCX_LargeAreaLowGrayLevelEmphasis×0.267+LAD_Kurtosis×0.230+LCX_Busyness×0.204+LCX_DependenceNonUniformityNormalized×0.177+RCA_SizeZoneNonUniformity×0.132+RCA_SizeZoneNonUniformityNormalized×0.119+RCA_Kurtosis×0.100+LCX_ClusterShade×0.037+RCA_ZoneVariance×0.015+LCX_ZoneEntropy×0.010-LCX_InverseVariance×0.016-RCA_HighGrayLevelZoneEmphasis×0.023-LAD_ClusterShade×0.056-LCX_DependenceEntropy×0.070-RCA_GrayLevelVariance×0.148-RCA_ClusterShade×0.154-LAD_Coarseness×0.232-LAD_LongRunHighGrayLevelEmphasis×0.233-LCX_SmallAreaEmphasis×0.242-RCA_ZoneEntropy×0.265-LCX_GrayLevelVariance×0.375

**Results**

**Table S1** Baseline characteristics of the training set and test set

| **Characteristic** | **Training set** | | | **Test set** | | | **p**** |
| --- | --- | --- | --- | --- | --- | --- | --- |
|  | Patient with CAD (n=102) | Patient without CAD (n=57) | p* | Patient with CAD (n=44) | Patient without CAD (n=26) | p* |  |
| Age (years) | 60.90 ± 8.84 | 56.28 ± 10.17 | 0.003 | 59.11 ± 9.38 | 55.46 ± 7.02 | 0.090 | 0.267 |
| Male sex, n (%) | 63 (61.8) | 30 (52.6) | 0.262 | 29 (65.9) | 17 (65.4) | 0.964 | 0.302 |
| Body mass index (kg/m^2^) | 26.15 [23.90; 27.70] | 25.80 [23.80; 28.15] | 0.876 | 25.27 ± 2.93 | 25.84 ± 3.93 | 0.462 | 0.284 |
| Obesity, n (%) | 21 (20.6) | 16 (28.1) | 0.284 | 7 (15.9) | 6 (23.1) | 0.669 | 0.428 |
| Smoking, n (%) | 27 (26.5) | 12 (21.1) | 0.446 | 8 (18.2) | 4 (15.4) | 1.000 | 0.216 |
| Drinking, n (%) | 16 (15.7) | 5 (8.8) | 0.217 | 5 (11.4) | 2 (7.7) | 0.934 | 0.495 |
| Hypertension, n (%) | 72 (70.6) | 31 (54.4) | 0.040 | 28 (63.6) | 11 (42.3) | 0.083 | 0.193 |
| Dyslipidaemia, n (%) | 16 (15.7) | 3 (5.3) | 0.052 | 6 (13.6) | 0 (0.0) | 0.127 | 0.450 |
| Family history, n (%) | 12 (11.8) | 2 (3.5) | 0.078 | 3 (6.8) | 3 (11.5) | 0.810 | 0.954 |
| Time since T2DM diagnosis (years) | 10.00 [4.00; 15.00] | 3.00 [0.00; 7.25] | <0.001 | 9.00 [2.00; 14.00] | 3.50 [1.00; 10.00] | 0.075 | 0.934 |
| Aspirin, n (%) | 6 (5.9) | 2 (3.5) | 0.781 | 3 (6.8) | 1 (3.8) | 1.000 | 1.000 |
| Statin, n (%) | 40 (39.2) | 21 (36.8) | 0.768 | 20 (45.5) | 12 (46.2) | 0.955 | 0.297 |
| Metformin, n (%) | 50 (49.0) | 25 (43.9) | 0.532 | 19 (43.2) | 14 (53.8) | 0.388 | 0.997 |
| Insulin, n (%) | 39 (38.2) | 28 (49.1) | 0.182 | 20 (45.5) | 9 (34.6) | 0.374 | 0.920 |
| Oral antihyperglycemic drugs, n (%) | 89 (87.3) | 44 (77.2) | 0.100 | 37 (84.1) | 25 (96.2) | 0.253 | 0.334 |
| HDL-cholesterol (mg/dL) | 0.99 [0.86; 1.10] | 1.01 [0.92; 1.21] | 0.082 | 0.95 [0.86; 1.11] | 1.02 [0.85; 1.11] | 0.784 | 0.617 |
| LDL-cholesterol (mg/dL) | 3.58 [3.16; 3.89] | 3.25 [2.74; 3.60] | 0.007 | 3.60 [3.02; 4.02] | 3.32 [3.15; 3.51] | 0.066 | 0.940 |
| Total cholesterol (mg/dL) | 5.52 [4.01; 6.28] | 4.65 [3.89; 5.80] | 0.085 | 5.15 ± 1.27 | 4.75 ± 1.16 | 0.193 | 0.549 |
| Triglyceride (mg/dL) | 0.84 [0.51; 2.84] | 2.07 [0.93; 2.98] | 0.014 | 0.79 [0.56; 2.55] | 2.10 [0.99; 2.71] | 0.066 | 0.525 |
| Fast glucose (mmol/L) | 10.15 [8.40; 12.60] | 8.30 [7.08; 11.23] | <0.001 | 10.30 [7.86; 13.10] | 8.95 [8.30; 11.30] | 0.481 | 0.708 |
| HbA1c (%) | 8.20 [7.40; 9.40] | 6.80 [6.30; 8.13] | <0.001 | 7.90 [7.20; 9.00] | 7.55 [6.50; 9.20] | 0.266 | 0.706 |
| Retinopathy, n (%) | 57 (55.9) | 27 (47.4) | 0.302 | 29 (65.9) | 19 (73.1) | 0.533 | 0.026 |
| Neuropathy, n (%) | 96 (94.1) | 53 (93.0) | 1.000 | 41 (93.2) | 25 (96.2) | 1.000 | 1.000 |
| Peripheral arterial occlusive disease, n (%) | 84 (82.4) | 41 (71.9) | 0.124 | 29 (65.9) | 21 (80.8) | 0.184 | 0.238 |
| Nephropathy, n (%) | 37 (36.3) | 21 (36.8) | 0.943 | 17 (38.6) | 8 (30.8) | 0.507 | 0.912 |
| CACS | 89.94 [16.60; 255.67] | 7.62 [1.00; 24.36] | <0.001 | 53.49 [15.16; 106.92] | 4.75 [1.78; 25.75] | <0.001 | 0.518 |
| PAT volume (mL) | 184.53 [145.56; 247.98] | 168.96 [126.34; 207.84] | 0.053 | 173.57 ± 67.32 | 192.23 ± 62.51 | 0.254 | 0.466 |
| CT-FFR | 0.75 [0.64; 0.85] | 0.91 [0.86; 0.94] | <0.001 | 0.76 [0.64; 0.85] | 0.90 [0.86; 0.93] | <0.001 | 0.831 |
| DS (%) | 54.00 [36.00; 73.00] | 28.00 [0.00; 52.00] | <0.001 | 60.50 [26.00; 69.00] | 27.50 [0.00; 52.00] | 0.002 | 0.878 |
| RCA-PCATA (HU) | -80.89 ± 8.90 | -87.88 ± 8.25 | <0.001 | -81.05 ± 8.80 | -93.85 ± 7.21 | <0.001 | 0.082 |
| LAD-PCATA (HU) | -82.03 ± 9.05 | -85.09 ± 7.89 | 0.034 | -79.91 ± 7.96 | -86.62 ± 7.03 | <0.001 | 0.557 |
| LCX-PCATA (HU) | -78.50 [-86.00; -72.00] | -83.00 [-88.25; -78.00] | <0.001 | -78.02 ± 7.94 | -85.27 ± 8.53 | <0.001 | 0.897 |

Values are mean ± standard deviation, median [25th and 75th percentile] or n (%). ***** p values reflect the differences between the patients with CAD and without CAD. **** p** values reflect the differences between the training set and test set.

*T2DM* type 2 diabetes mellitus, *HDL* high-density lipoprotein, *LDL* low-density lipoprotein, *HbA1c* glycated haemoglobin, *CACS* coronary artery calcium score, *PAT* pericardial adipose tissue, *CT-FFR* computed tomography-based fractional flow reserve, *DS* diameter stenosis, *PCATA* pericoronary adipose tissue attenuation

**Table S2** Pairwise comparison of the AUCs of the test set by the DeLong test

|  | 95%CI | p |
| --- | --- | --- |
| Model 1 vs. Model 2 | 0.129 – 0.350 | <0.001 |
| Model 1 vs. Model 3 | -0.003 – 0.009 | 0.382 |
| Model 1 vs. Model 4 | 0.129 – 0.348 | <0.001 |
| Model 2 vs. Model 3 | 0.127 – 0.346 | <0.001 |
| Model 2 vs. Model 4 | -0.005 – 0.007 | 0.776 |
| Model 3 vs. Model 4 | 0.128 – 0.344 | <0.001 |

*AUC* area under the curves, *95%CI* 95% confidence interval, *Model 1* clinical factors model, *Model 2* clinical factors and imaging indexes model, *Model 3* clinical factors and Radscore model, *Model 4* combined model

**Table S3** Comparison of CT parameters in patients with T2DM with significant coronary stenosis

|  | Patient with CAD | Patient without CAD | p |
| --- | --- | --- | --- |
| n | 86 | 22 |  |
| CACS | 125.67 [39.78; 291.75] | 9.74 [0.91; 47.78] | <0.001 |
| PAT volume (mL) | 199.73 ± 71.75 | 182.59 ± 75.42 | 0.325 |
| CT-FFR | 0.68 [0.61; 0.75] | 0.86 [0.81; 0.88] | <0.001 |
| DS (%) | 69.00 [61.00; 83.00] | 59.50 [55.00; 68.00] | 0.001 |
| RCA-PCATA (HU) | -80.50 [-86.00; -74.00] | -87.00 [-91.00; -82.00] | <0.001 |
| LAD-PCATA (HU) | -80.74 ± 7.62 | -84.59 ± 5.51 | 0.029 |
| LCX-PCATA (HU) | -76.00 [-85.00; -72.00] | -85.00 [-90.00; -80.00] | <0.001 |

Values are mean ± standard deviation, median [25th and 75th percentile] or n (%). p values signifies statistical significance and reflect the differences between patients with and without CAD.

*T2DM* type 2 diabetes mellitus, *CAD* coronary artery disease*, CACS* coronary artery calcium score, *PAT* pericardial adipose tissue, *CT-FFR* computed tomography-based fractional flow reserve, *DS* diameter stenosis, *PCATA* pericoronary adipose tissue attenuation

**Table S4** Comparison of CT parameters in patients with T2DM without significant coronary stenosis

|  | Patient with CAD | Patient without CAD | p |
| --- | --- | --- | --- |
| n | 60 | 61 |  |
| CACS | 36.17 [5.10; 107.75] | 7.08 [1.73; 15.53] | <0.001 |
| PAT volume (mL) | 163.35 [125.23; 230.09] | 173.55 [132.51; 209.66] | 0.909 |
| CT-FFR | 0.85 [0.79; 0.89] | 0.92 [0.90; 0.95] | <0.001 |
| DS (%) | 29.00 [15.00; 42.50] | 4.00 [0.00; 32.25] | <0.001 |
| RCA-PCATA (HU) | -82.87 ± 9.22 | -90.39 ± 8.30 | <0.001 |
| LAD-PCATA (HU) | -82.32 ± 10.17 | -85.92 ± 8.26 | 0.035 |
| LCX-PCATA (HU) | -80.07 ± 7.91 | -83.71 ± 7.49 | 0.011 |

Values are mean ± standard deviation, median [25th and 75th percentile] or n (%). p values signifies statistical significance and reflect the differences between patients with and without CAD.

*T2DM* type 2 diabetes mellitus, *CAD* coronary artery disease*, CACS* coronary artery calcium score, *PAT* pericardial adipose tissue, *CT-FFR* computed tomography-based fractional flow reserve, *DS* diameter stenosis, *PCATA* pericoronary adipose tissue attenuation

**Table S5** Comparison of CT parameters in patients with CAD with and without significant stenosis

|  | Patient with significant stenosis | Patient without significant stenosis | p |
| --- | --- | --- | --- |
| n | 86 | 60 |  |
| CACS | 125.67 [39.78; 291.75] | 36.17 [5.10; 107.75] | <0.001 |
| PAT volume (mL) | 185.47 [149.56; 244.80] | 163.35 [125.23; 230.09] | 0.102 |
| CT-FFR | 0.68 [0.61; 0.75] | 0.85 [0.79; 0.89] | <0.001 |
| DS (%) | 69.00 [61.00; 83.00] | 29.00 [15.00; 42.50] | <0.001 |
| RCA-PCATA (HU) | -80.50 [-86.00; -74.00] | -83.00 [-88.00; -79.00] | 0.020 |
| LAD-PCATA (HU) | -80.74 ± 7.62 | -82.32 ± 10.17 | 0.288 |
| LCX-PCATA (HU) | -77.71 ± 8.07 | -80.07 ± 7.91 | 0.082 |

Values are mean ± standard deviation, median [25th and 75th percentile] or n (%). p values signifies statistical significance and reflect the differences between patients with and without significant stenosis.

*CAD* coronary artery disease*, CACS* coronary artery calcium score, *PAT* pericardial adipose tissue, *CT-FFR* computed tomography-based fractional flow reserve, *DS* diameter stenosis, *PCATA* pericoronary adipose tissue attenuation


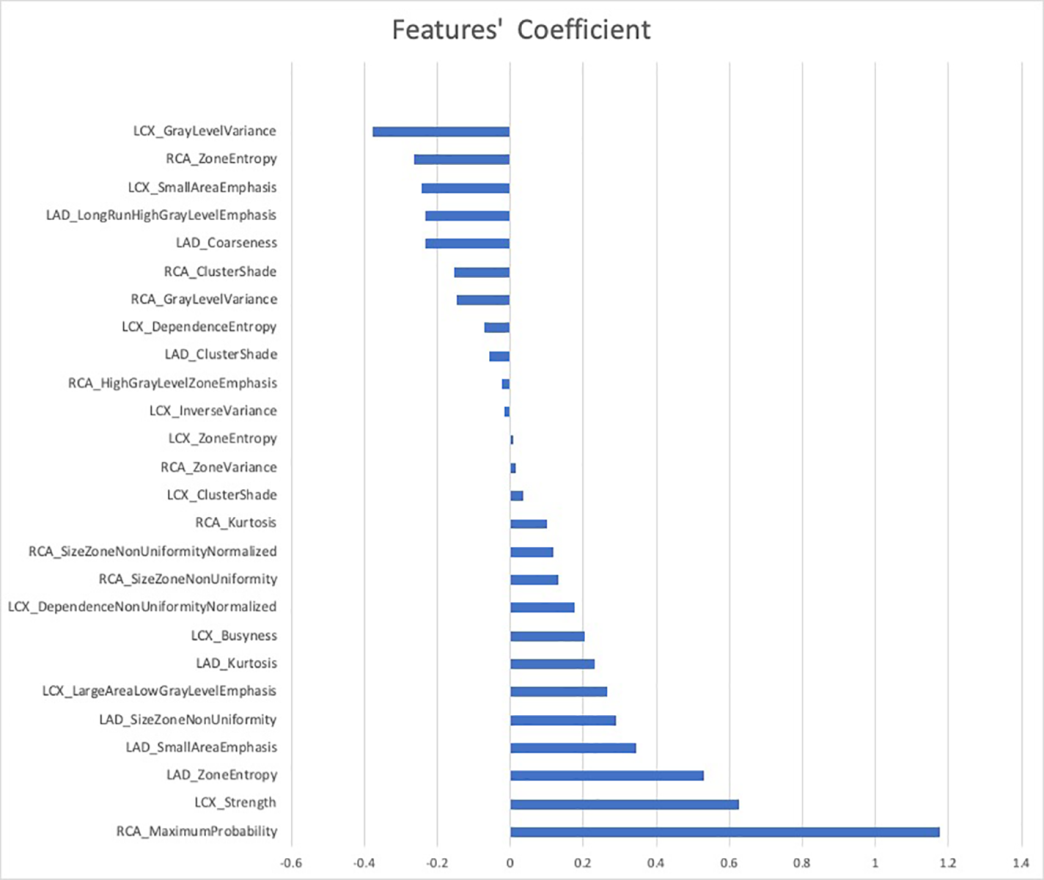


**Fig. S1** The relative importance of radiomics features.
